# Physical rehabilitation interventions for adult intensive care patients across the continuum of recovery: an overview of systematic reviews

**DOI:** 10.1186/1745-6215-16-S2-P160

**Published:** 2015-11-16

**Authors:** Bronwen Connolly, Kathryn McDowell, Brenda O'Neill, Lisa Salisbury, Bronagh Blackwood

**Affiliations:** 1 Guy's and St.Thomas’ NHS Foundation Trust, London, UK; 2 King's College London, London, UK; 3 Ulster University, Ulster, UK; 4 University of Edinburgh, Edinburgh, UK; 5 Queen's University Belfast, Belfast, UK

## Background

Patients admitted to the intensive care unit (ICU) with critical illness often experience significant physical impairments, which typically persist for many years following resolution of the original illness. Physical rehabilitation interventions that enhance restoration of physical function have been evaluated across the continuum of recovery following critical illness including within the ICU, following discharge to the ward, and beyond hospital discharge. Multiple systematic reviews have been published appraising the expanding evidence investigating such physical rehabilitation interventions, although there appears to be variability in review methodology and quality.

## Objective

To conduct an overview of existing systematic reviews of physical rehabilitation interventions for adult intensive care patients across the continuum of recovery.

## Methods

This protocol is registered on PROSPERO (CRD 42015001068). We will search the Cochrane Systematic Review Database, Database of Abstracts of Reviews of Effectiveness, Cochrane Central Register of Controlled Trials, MEDLINE, EMBASE, and CINAHL. We will include systematic reviews of randomised controlled trials of adult patients, admitted to the ICU, and who have received physical rehabilitation interventions at any timepoint during their recovery. Data extraction will include eligible systematic review aims and rationale, study types, populations, interventions, comparators, outcomes and quality appraisal method. Quality of reporting and methodological quality will be appraised using the PRISMA checklist, and the AMSTAR tool.

## Discussion

We anticipate the findings from this novel overview of systematic reviews will contribute to the synthesis and interpretation of existing evidence regarding physical rehabilitation interventions and physical recovery in post critical illness patients across the continuum of recovery.

